# Challenge to Eliminate Parasite Palatal Infestation in a Deaf–Mute Geriatric Patient

**DOI:** 10.1155/crid/5581576

**Published:** 2025-10-30

**Authors:** Amelia Elizabeth Pranoto, Nina Runting

**Affiliations:** ^1^Oral and Maxillofacial Surgeon Department at Dentistry Faculty, Universitas Muhammadiyah Surakarta, Surakarta, Indonesia; ^2^Oral and Maxillofacial Surgeon at Public Hospital Wates Regency, Daerah Istimewa Yogyakarta, Indonesia; ^3^Oral and Maxillofacial Surgeon at Dental Hospital Soelastri, Surakarta, Indonesia

**Keywords:** disability, geriatric, maggot infestation, oral myiasis, pain, periodontal disease

## Abstract

Myiasis is a human parasitic disease caused by fly larvae that is rarely found in the oral cavity. Poor oral hygiene among older males can lead to intraoral myiasis. This report describes a rare case of severe, atypical pain caused by maxillary periodontal myiasis in an elderly disabled patient. Maggots infested the hard palate near the anterior maxillary teeth. Despite a radiological examination showing no significant hard tissue alterations, numerous maggots were manually extracted from a hole in the anterior hard palate. Surgical tooth extraction and maggot removal were both performed under general anaesthesia. The patient was also given albendazole to eliminate any remaining larvae.

## 1. Introduction

Myiasis is a parasitic disease caused by the deposition of fly larvae in tissues; its involvement in the human oral cavity, however, is uncommon [1, 2]. Although the term myiasis was coined in 1840, it has been known since ancient times. Myiasis has been extensively documented in humans since the early 20th century, and the condition is more prevalent in tropical and subtropical regions. Oral myiasis is more common in elderly males who have low income, medical comorbidities, neglected wounds and poor oral hygiene [3–5]. Dos Passos et al. reported that most countries that reported oral myiasis were India and Brazil [2].

Most myiasis cases are caused by fly larvae, such as dipterous larvae (maggots), *Chrysomya bezziana* and *Wohlfahrtia magnifica* [6–8]. The majority of flies responsible for myiasis can be classified into two categories based on their association with their hosts. Obligate parasites proliferate exclusively within the healthy tissues of living hosts. Conversely, facultative parasites, typically associated with carrion, faeces or decomposing plant matter, can proliferate in the necrotic tissue of living organisms and generally refrain from invading healthy tissue [9]. Accidental infestations constitute a distinct category where eggs or larvae of saprophagous flies are unintentionally breathed or ingested alongside contaminated food, resulting in gastrointestinal myiasis [10].

The prevalence of oral myiasis is significantly lower than that of cutaneous myiasis, as oral tissues are not continuously exposed to the external environment [11]. Intraoral myiasis can occur when flies are attracted to malodorous conditions within the mouth, typically resulting from poor oral hygiene or decomposing food remnants. Continuous mouth opening allows adult flies to lay their eggs. Adult females oviposit in viviparous animals (including humans). Infestation sites typically consist of superficial wounds, open sores and mucous membranes located in bodily orifices, including the mouth, ear and nose. The eggs were incubated for 24 h after the larvae penetrated the host tissues headfirst into the incision in a distinctive screw-like manner, consuming the living tissue. Larvae emit toxins that degrade host tissues. Proteolytic enzymes secreted by adjacent bacteria degrade the tissues on which the larvae subsist. The larvae developed within 5–7 days. They then extricate themselves from the wound and descend to the ground to pupate [7, 8].

Oral and maxillofacial surgeons primarily treat patients with myiasis. Sankari and Ramakrishnan used turpentine oil, followed by the manual removal of maggots using tweezers. Antibiotics such as amoxicillin, clavulanic acid and ibuprofen with paracetamol were prescribed [8]. Shenoi et al. also used turpentine oil to attract maggots from tissue cavities and then extracted them using curved artery forceps for 5 consecutive days. Hydrogen peroxide, povidone–iodine and metronidazole were used to irrigate the wounds after deep exploration and curettage. The patient was administered ceftriaxone, pantoprazole and metronidazole for 5 days. No attempt was made to administer the antiparasitic drug ivermectin [7].

Each physician has their treatment preference based on familiarity and the availability of the drug materials. In Indonesia, we do not use a suffocating agent as a medication. Additionally, the Inventory Multi-Tiered Assessment and Prioritisation classified turpentine oil as hazardous, causing acute toxicity upon inhalation, contact with the skin, ingestion or entry into the airways, as well as allergic skin reactions [12]. Although several case reports have used turpentine oil to suffocate maggots before extraction, the author identified gaps in these treatments due to the lack of resource availability (turpentine oil), patient safety concerns and insufficient guidelines owing to its rarity. This could lead practitioners to treat oral myiasis without proper guidance.

We report a rare case of palatal periodontal myiasis in an elderly Indonesian male with a disability. We also present approaches based on evidence-based medicine and rationalisation using pathophysiological theories of parasitic infestation.

## 2. Case

### 2.1. Ethical Statement

Written informed consent was obtained from the patient's guardian prior to the study.

### 2.2. Subjective and Objective Examination

**Figure 1:**
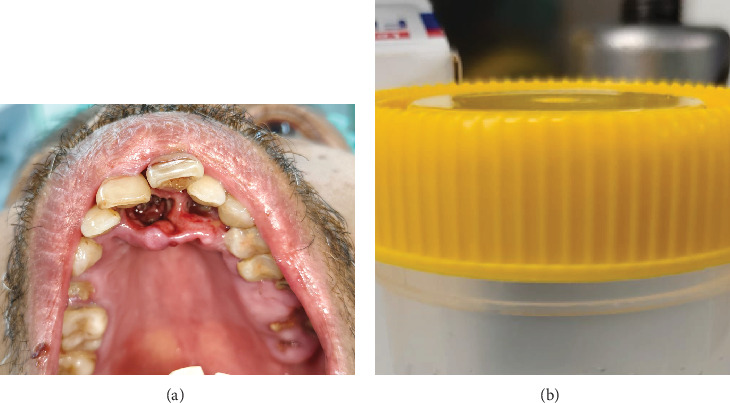
(a) Clinical picture of periodontal tissue in the anterior hard palate filled with maggots. (b) Extracted maggots after surgery in a container filled with alcohol 70%.

**Figure 2:**
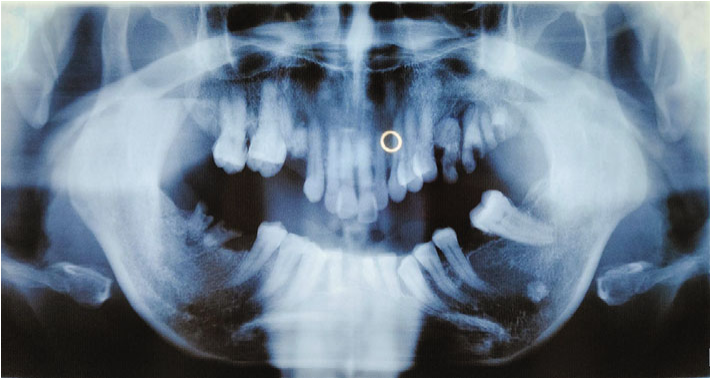
An orthopantomogram revealed slight alveolar bone resorption around the interdental Teeth 11, 12, 21 and 22, as well as multiple Dental Root Teeth 15, 26, 27, 28 and 47.

A 69-year-old male presented to the Oral and Maxillofacial Department of Wates Public Hospital because of atypical, intense pain in his upper jaw that had persisted for almost 3 months. The patient came from a remote area and sought medical treatment near his residence. Oral antibiotics and analgesics were prescribed; however, the condition persisted. The patient, who was deaf, mute, illiterate and lived alone, communicated with the doctor through gestures, with most of the conversation translated by his acquaintance (a neighbour). He reported no history of systemic diseases and had never been hospitalised. The extraoral clinical appearance was normal, and the lymph nodes were not swollen. A strong intraoral malodour and protruding maxillary anterior teeth were observed. The doctor found a necrotic wound in the palate that was filled with maggots (Figure 1). The anterior upper teeth were only slightly luxated, and multiple dental roots were present in both jaws. Slight erythema, mucosal oedema and numerous hollows in the anterior palate were observed. The patient had no history of trauma but regularly breathed through his mouth due to lip incompetence. The patient also stated that he periodically brushed his teeth at night. Radiological examination (orthopantomogram) revealed no extensive bone necrosis, only slight alveolar bone resorption around the Interdental Teeth 11, 21 and 22 and multiple Dental Root Teeth 15, 26, 27, 28 and 47 (Figure 2).

### 2.3. Diagnosis and Aetiology

Based on subjective and objective assessments, the diagnosis was primary oral myiasis of the maxillary anterior palate. In this case, the infestation was attributed to protrusion of the maxillary anterior teeth, periodontal tissue inflammation and insufficient oral hygiene, which was characterised by malodour and dental calculus.

.

## 3. Case Management

### 3.1. Treatment Objective

The oral and maxillofacial surgeon planned to remove all maggots, surgically clean the area and extract Teeth 11, 12, 21 and 22 while cleaning all dental roots to prevent further infection under general anaesthesia.

### 3.2. Treatment Alternatives

General anaesthesia was chosen to enable complete debridement and ensure patient comfort during surgery. Suffocating agents were not used because the lesion was accessible for surgical treatment, and the agents were unavailable. The patient was administered prophylactic antibiotics (cefazolin 2 g) 30 min prior to surgery. Postoperatively, once the patient regained full consciousness and demonstrated adequate oral intake, oral antihelminthic medication (albendazole tablets [Kimia Farma], 400 mg three times daily), antibiotics (intravenous metronidazole [OGB Dexa], 500 mg three times daily) and analgesics (intramuscular ketorolac [OGB Dexa], 30 mg three times daily) were prescribed. The patient was discharged after 5 days of hospitalisation and postoperative wound management. Follow-up was conducted after 1 and 6 months, as the patient resided alone and relied entirely on his neighbour for hospital visits. The patient reported no complaints, and there was no evidence of maggot infestation in the periodontal tissue after the surgery.

## 4. Discussion

Maggots infested the palatal periodontal tissue of this senior, disabled male due to his protrusive maxillary anatomical contour, poor oral hygiene and mouth breathing. Even though he did not have severe periodontitis in the palatal periodontal tissue, the foul odour from poor oral hygiene could attract flies to lay eggs on the palatal mucosa [10, 13]. The patient was disabled and lived alone, which was a significant reason for his poor hygiene. Extensive plaque and calculus were observed on the tooth surfaces. Several teeth were decayed in both the upper and lower jaws. These larvae feed on living or dead host tissue, liquid body substances, undigested food or food retained on the tooth surface [14].

The larvae hatch in approximately 8–10 h, after which they burrow into the surrounding tissues, causing tissue inflammation, causing discomfort and prompting the patient to consult a doctor [15]. In this case, the patient reported atypical yet severe maxillary pain. As the patient was mute and deaf, the previous dentist had trouble communicating and failed to identify the source of his complaint. We hypothesised that the patient experienced intense pain because the maggot infestation caused severe inflammation of the palatal periodontal tissue. Preoperative clinical features revealed redness of the anterior palatal mucosa with moderately swollen tissue.

The patient underwent surgery under general anaesthesia. An oral and maxillofacial surgeon extracted Teeth 11, 12, 21 and 22, along with all maggots that had infested the hole in the anterior hard palate. This method is effective and safe, allowing complete maggot removal and thorough debridement of necrotic tissue with minimal bleeding or pain. Gupta Vinit et al. [16], Shukla et al. [17] and De Souza et al. [18] removed all maggots from the mouth under general anaesthesia. The larvae can burrow deep into the underlying, inaccessible areas and attach to the tissue cavity. Such conditions render mechanical removal under local anaesthesia impossible [17, 18].

Topical asphyxiating chemicals, including ether, chloroform, turpentine oil, petroleum jelly and eucalyptus oil, function as irritants, compelling aerobic organisms to ascend in pursuit of oxygen so that they can be extracted using forceps or tweezers. A comparable method was employed in the aforementioned case due to the dissemination of larvae into deep, inaccessible regions of the oral cavity [18]. This case was managed under general anaesthesia without the use of asphyxiating medications, as the maggots were localised on the hard palate, and the patient's communication and intellectual limitations precluded invasive procedures while conscious. Following sedation under general anaesthesia, the maggots were manually extracted through a broad mucoperiosteal flap, which provided an adequate surgical field. The decision to perform thorough debridement and larval extraction under general anaesthesia was based on the patient's disability and the impracticality of frequent hospital visits, given his solitary residence in a remote area distant from the hospital. The use of asphyxiating chemicals would have required multiple clinic visits until complete wound healing and closure, which was impractical given the patient's remote living situation and dependence on neighbours for access to hospitals.

Albendazole has been clinically proven as an effective adjuvant therapy for maggot infestations, alongside surgery. Sunny et al. and Patel et al. used oral albendazole 400 mg twice a day for 5 days to treat oral myiasis after surgery had been done. Albendazole is a broad-spectrum antihelminthic–antiparasitic agent and has been used for the treatment of intestinal myiasis and cutaneous myiasis [19]. In this case, albendazole was effective in treating the maggot infestation, with no recurrence observed after 6 months of follow-up. Furthermore, it is important to consider that other recently introduced compounds have a significant influence on the oral environment. The application of ozonised gels [20] and probiotics [21] warrants consideration in future investigations to ascertain their potential synergistic effects with albendazole on tissue healing.

The limitations of this case report are the absence of clinical photographs during the patient's follow-up visits at 1 and 6 months postoperatively. The tooth extraction performed in this case may not be universally applicable to all cases of oral myiasis because of the varying contributing factors in each case. In this patient, extraction was carried out to prevent recurrent maggot infection due to the inability of the mouth to close tightly, caused by the protrusion of the maxillary anterior teeth and severe periodontal tissue infection in the maxillary anterior area resulting from crowded teeth and poor oral hygiene.

## 5. Conclusion

Myiasis is an infrequent parasitic infection in humans, predominantly affecting older adults and individuals with disabilities. Formulating flexible guidelines for treating myiasis is essential, as they would also be applicable in remote areas that lack medical supplies. Public education regarding the risks of oral myiasis is essential; also, we have to raise caregiver awareness to maintain thorough oral hygiene and encourage routine dental check-ups every 6 months.

## Data Availability

The data that support the findings of this study are available from the corresponding author upon reasonable request.

## References

[1] Francesconi F., Lupi O. (2012). Myiasis. *Clinical Microbiology Reviews*.

[2] Dos Passos J. B. S. (2021). Oral Myiasis: Analysis of Cases Reported in the English Literature From 1990 to 2020. *Special Care in Dentistry*.

[3] Taş Cengiz Z., Yılmaz H., Beyhan Y. E., Yakan Ü., Ekici A. (2019). An Oral Myiasis Case Caused by Diptera (Calliphoridae) Larvae in Turkey. *Türkiye Parazitolojii Dergisi*.

[4] Ali F. M., Patil K., Kar S., Patil A. A., Ahamed S. (2016). Oral Myiasis Affecting Gingiva in a Child Patient: An Uncommon Case Report. *Case Reports in Dentistry*.

[5] Meurer M. I., Grando L. J., Rivero E. R., Souza C. E., Marcondes C. B. (2016). A Rare Case of Labial Myiasis Caused by Dermatobia hominis. *The Journal of Contemporary Dental Practice*.

[6] Singh S. P., Prashar S., Kajla P., Hashmi Z., Gowda S. (2024). Oral Myiasis—An Uncommon Finding in Modern Era. *Journal of Maxillofacial and Oral Surgery*.

[7] Shenoi R., Kolte V., Ingole P. (2020). Management of Oral Myiasis Caused by *Chrysomya bezziana* - A Case Series. *Annals of Maxillofacial Surgery*.

[8] Sankari L. S., Ramakrishnan K. (2010). Oral Myiasis Caused by Chrysomya bezziana. *Journal of Oral and Maxillofacial Pathology*.

[9] Sunny B., Sulthana L., James A., Sivakumar T. (2016). Maggot Infestation: Various Treatment Modalities. *The Journal of the American College of Clinical Wound Specialists*.

[10] Caissie R., Beaulieu F., Giroux M., Berthod F., Landry P. É. (2008). Cutaneous Myiasis: Diagnosis, Treatment, and Prevention. *Journal of Oral and Maxillofacial Surgery*.

[11] Rossi-Schneider T., Cherubini K., Yurgel L. S., Salum F., Figueiredo M. A. (2007). Oral Myiasis: A Case Report. *Journal of Oral Science*.

[12] (2018). Inventory Multi-Tiered Assessment and Prioritisation. Turpentin: Human Health Tier II Assessment. IMAP Group Assessment Report. https://www.nicnas.gov.au/chemical-information/imap-assessments/imap-group-assessment-report?assessment_id=12643.

[13] Aswath N. (2022). Oral Myiasis. *The Pan African Medical Journal*.

[14] Kumar P., Singh V. (2014). Oral Myiasis: Case Report and Review of Literature. *Oral and Maxillofacial Surgery*.

[15] Pereira T., Tamgadge A. P., Chande M. S., Bhalerao S., Tamgadge S. (2010). Oral Myiasis. *Contemporary Clinical Dentistry*.

[16] Vinit G. B. G., Jayavelu P., Shrutha S. P. (2013). Oral Myiasis in a Maxillofacial Trauma Patient. *Journal of Pharmacy & Bioallied Sciences*.

[17] Shukla A. D., Kamath A. T., Kudva A., Pai D., Patel N. (2016). Our Experience in the Management of Traumatic Wound Myiasis: Report of 3 Cases and Review of the Literature. *Case Reports in Dentistry*.

[18] De Souza N., Kamat S., Chalakkal P., Da Costa G. C. (2018). A Rare Occurrence of Oral Myiasis in the Posterior Region of the Jaw. *International Journal of Contemporary Medical Research*.

[19] Patel B. C., Ostwal S., Sanghavi P. R., Joshi G., Singh R. (2018). Management of Malignant Wound Myiasis With Ivermectin, Albendazole, and Clindamycin (Triple Therapy) in Advanced Head-and-Neck Cancer Patients: A Prospective Observational Study. *Indian Journal of Palliative Care*.

[20] Colombo M., Gallo S., Garofoli A., Poggio C., Arciola C. R., Scribante A. (2021). Ozone Gel in Chronic Periodontal Disease: A Randomized Clinical Trial on the Anti-Inflammatory Effects of Ozone Application. *Biology*.

[21] Henrique Soares K., Firoozi P., Maria de Souza G., Beatriz Lopes Martins O., Gabriel Moreira Falci S., Rocha Dos Santos C. R. (2023). Efficacy of Probiotics Compared to Chlorhexidine Mouthwash in Improving Periodontal Status: A Systematic Review and Meta-Analysis. *International Journal of Dentistry*.

